# Thermal Stress Response Profiling Reveals Adaptive Advantages of Indigenous Hercegovačka and Dubska Pramenka Sheep

**DOI:** 10.3390/ani15182678

**Published:** 2025-09-13

**Authors:** Husein Ohran, Naris Pojskic, Jasmin Ramic, Szilvia Kusza, Naida Lojo-Kadric, Aida Hodzic

**Affiliations:** 1 Department of Basic Veterinary Sciences, University of Sarajevo–Veterinary Faculty, Zmaja od Bosne 90, 71000 Sarajevo, Bosnia and Herzegovina; aida.hodzic@vfs.unsa.ba; 2 Institute for Genetic Engineering and Biotechnology, University of Sarajevo, Zmaja od Bosne 8, 71000 Sarajevo, Bosnia and Herzegovina; naris.pojskic@ingeb.unsa.ba (N.P.); jasmin.ramic@ingeb.unsa.ba (J.R.); naida.lojo@ingeb.unsa.ba (N.L.-K.); 3 Centre for Agricultural Genomics and Biotechnology, Faculty of Agricultural and Food Sciences and Environmental Management, University of Debrecen, Egyetem tér 1, 4032 Debrecen, Hungary; kuszasz@hotmail.com

**Keywords:** climate resilience, heat shock proteins, gene expression, indigenous sheep breeds, thermal adaptation

## Abstract

Climate change poses an increasing challenge to livestock production, making the identification of thermally resilient breeds crucial. This study investigated the expression patterns of eight thermal stress-related genes (HSP90AA1, HSPA8, HSPA1A, IL-6, IL-10, TNF-α, NOS-3, and SOD-2) in two strains of indigenous Pramenka sheep (Hercegovačka and Dubska) under different climatic conditions. Blood samples were collected from 96 sheep across four locations during summer and winter seasons. Gene expression was analyzed using RT-PCR and evaluated against comprehensive environmental data, including the temperature–humidity index (THI). Principal component analysis revealed distinct gene expression patterns between strains and seasons. The Hercegovačka strain showed consistently higher expression of HSP90AA1 compared to the Dubska strain in both seasons (summer: 5.81 vs. 2.47; winter: 4.38 vs. 1.19, *p* < 0.05), with all individuals exhibiting upregulation during summer and 95.8% showing upregulation during winter. Both strains demonstrated reduced expression of pro-inflammatory cytokines (IL-6, TNF-α) and increased expression of anti-inflammatory IL-10, suggesting maintained immunocompetence under thermal stress. The expression patterns of oxidative stress-related genes (SOD-2, NOS-3) indicated effective management of oxidative stress in both strains. These results suggest superior thermal adaptation in the Hercegovačka strain, particularly through HSP-mediated cellular protection mechanisms, while both strains show favorable immune and oxidative stress responses. These findings provide valuable insights for climate-resilient sheep breeding programs and the conservation of adaptive genetic resources.

## Simple Summary

Climate change is becoming a serious threat to animal farming, making it important to find animals that can cope well with high and low temperatures. This study focused on two strains of indigenous Pramenka sheep from Bosnia and Herzegovina to determine how they respond to heat and cold. Researchers looked at the activity of specific genes that help animals deal with temperature stress using blood samples from 96 sheep collected in summer and winter across different regions. The results showed that one strain, Hercegovačka sheep, had much higher activity of a gene that protects cells from heat damage, especially during hot weather. Both strains showed strong abilities to control inflammation and protect themselves from harmful effects caused by heat, which helps them stay healthy in challenging climates. This study concluded that Hercegovačka sheep have better natural protection against temperature changes, but both strains have useful traits for surviving in a changing climate. These results can help farmers and scientists choose and protect sheep breeds that are more likely to thrive as weather conditions become more extreme, ensuring food production and supporting rural communities in the future.

## 1. Introduction

Livestock production faces significant challenges due to climate change, threatening both animal welfare and economic sustainability. Studies have shown that genetic diversity, as a key component of biodiversity alongside ecosystems and species, is essential for animals to adapt to climatic changes [1]. In sheep populations, this genetic diversity is particularly important for thermal adaptation mechanisms.

Recent morphometric and genetic analyses have provided valuable insights into sheep breed differentiation and adaptation. Markovic et al. [2] conducted a comprehensive study comparing morphometric characteristics across six breeds of Pramenka sheep, finding significant differentiation in body measurements and proportions that reflected adaptation to different environmental conditions. Their research revealed that breeds like Sjenicka had larger body sizes suitable for mountain conditions, while breeds like Zeta Žuja developed smaller frames adapted to Mediterranean climates. Complementing these morphological studies, genetic analyses using microsatellite markers have revealed the underlying population structure of Pramenka sheep breeds. Salamon et al. [3] examined 12 eastern Adriatic and western Dinaric sheep breeds, documenting considerable genetic diversity within populations and moderate differentiation between breeds. Their findings showed that, while some breeds maintained distinct genetic profiles, others demonstrated evidence of admixture, likely reflecting historical breeding practices and geographical proximity.

When ambient temperature exceeds the upper or lower critical values of domestic animals’ internal temperature, thermal stress occurs [4]. According to Van Wettere et al. [5], sheep experience negative effects when the temperature falls below 12 °C (lower critical temperature) or rises above 25 to 31 °C (upper critical temperature). Although sheep are generally considered resistant to harsh environments, thermal stress negatively affects both physiology [6] and productive performance [7].

The impact of thermal stress operates at multiple biological levels. At the molecular level, it can directly cause protein denaturation and alter cellular metabolism, leading to increased reactive oxygen species (ROS) production, which can damage DNA and membrane lipids [8]. Moreover, thermal stress suppresses the immune response through the activation of the hypothalamic–pituitary–adrenal and sympathetic–adrenal–medullary axes, consequently affecting immunological reactions [9].

A recent study by Wanjala et al. [10] highlights the inherent ability of indigenous sheep breeds to withstand the effects of climate change, identifying them as vital resources for breeding climate-resilient traits. This adaptation capacity has significant economic implications for grassland-based livestock systems, where forage quality and availability directly influence production costs and animal performance [11]. As stated by FAO [12], locally adapted breeds represent valuable genetic resources, not only for their production potential but more importantly for their resilience and adaptability to harsh environmental conditions, including climate extremes and low-input farming systems, contributing to both the sustainability of livestock production and the preservation of biodiversity. Studies on Hungarian Tsigai sheep have shown them to be particularly well-adapted to continental climate conditions, with excellent thermotolerance and immunity [13]. These adaptations are reflected in their genetic diversity, as revealed by microsatellite analysis [14] and more recent SNP marker studies. Similarly, research on Montenegrin sheep populations has demonstrated their genetic distinctness and adaptation to different climatic regions [15].

The most common indigenous animal breed in Bosnia and Herzegovina is Pramenka sheep. Pramenka is considered extremely resistant and adaptable. Studies using microsatellite and mitochondrial DNA markers have revealed significant genetic diversity within this group [16], suggesting their potential value for climate resilience breeding programs. Their wide geographical distribution throughout the Balkan Peninsula, particularly in remote and challenging environments, attests to their remarkable adaptability. Although their overall population appears high, suggesting that the breed is not globally endangered, extensive and uncontrolled cross-breeding with more productive foreign breeds has significantly reduced the availability of genetically pure individuals. The two targeted Pramenka strains, with their populations being sparsely distributed and increasingly fragmented across their traditional regions, are considered endangered.

One of the main hallmarks of thermal stress at the cellular level is the dramatic alteration in gene expression patterns. In recent years, several studies have employed molecular techniques and have identified various genes associated with thermal adaptation in sheep [17,18,19]. Key heat stress resistance genes in sheep include those involved in cellular stress response (such as HSP70 and HSP90AA1), oxidative stress defense (SOD3, GPX1), thermoregulation and metabolic homeostasis (ATP1A1, ENPP1), as well as immune response (IL10, TNF-α, TLR4) and production and reproduction traits (LEP, GH, IGF-1, FSHR). However, many studies are often limited by small sample sizes, a focus on commercial rather than indigenous breeds, and insufficient integration of environmental and physiological data. Understanding the expression patterns of these genes under different environmental conditions can provide valuable insights into adaptive mechanisms.

Heat shock proteins, particularly the HSP70 family, are well-documented for their role during thermal stress, acting as molecular chaperones that aid in maintaining protein integrity under unfavorable conditions [20]. HSPs are upregulated in response to increased temperatures, which is pivotal in cellular protection and maintenance of homeostasis. For instance, in earlier studies involving goats, thermal stress led to a notable rise in the expression of HSP genes, showcasing their protective role against cellular damage [21]. Furthermore, the expression of HSPs can be correlated with immune responses. Research has shown that HSPs influence the expression of various immune-related genes, thus providing a layer of resilience to the organism during periods of stress [22,23]. According to a study, the overexpression of HSP72, a critical member of the HSP family, plays a protective role against oxidative stress, highlighting their integration with immune mechanisms [24]. In sheep, leukocyte counts were influenced by thermal stress, showing that immune responses are modulated during heightened temperatures, corroborating that HSPs may facilitate such responses [25]. Additionally, oxidative stress is a crucial factor in the adverse effects of thermal stress. HSPs interact with oxidative stress-related pathways, effectively mitigating the effects of ROS generated during thermal exposure. The expression of antioxidant enzymes, such as superoxide dismutase (SOD), is often regulated in conjunction with HSP expression, providing a comprehensive defense against the oxidative stress that accompanies elevated temperatures [26].

Our hypothesis was that indigenous Pramenka sheep strains (Hercegovačka and Dubska) would exhibit distinct expression patterns of thermal stress-related genes under different climatic conditions, reflecting strain-specific adaptive capabilities. We hypothesized that these breeds, having evolved in harsh environments on the Balkan Peninsula, would demonstrate superior molecular mechanisms for thermal adaptation through upregulation of heat shock proteins, modulation of immune-related cytokines, and also effective regulation of oxidative stress genes, with potential differences between strains indicating varying degrees of thermal resilience.

The present study aims to evaluate the expression of key thermal stress-related genes in different strains of Pramenka sheep to understand the molecular basis of their environmental adaptation. This research contributes to our understanding of thermal stress response mechanisms and the development of long-term strategies for the genetic conservation of indigenous breeds. By informing future breeding programs aimed at enhancing climate resilience, the results support efforts to preserve valuable adaptive traits while maintaining genetic purity within local sheep populations.

## 2. Materials and Methods

### 2.1. Animals and Experimental Design

The research covered two strains of Pramenka sheep, namely Dubska and Hercegovačka. To reduce the risk of inbreeding, sampling was conducted across two different farms, each located in two different areas for each of the investigated strains: mountains Vlašić and Kupres for the Dubska strain and the area Podveležje and the town of Nevesinje for the Hercegovačka strain. The sampling process involved the same sheep, identified either by ear tags or other means of identification (head color, height to the withers, etc.), during two periods: high temperatures (July 2020) and low temperatures (February 2020). The animal selection process followed a two-staged approach: firstly, farms were randomly selected within each location, followed by the random selection of individual animals within the chosen farm. All sheep were non-pregnant females, aged between 2 and 4 years. Twelve blood samples were collected from each locality and during each sampling period, resulting in a total of 24 samples per strain across both summer and winter periods, equating to 48 samples for each of the two examined strains. Consequently, the overall number of sheep blood samples amounted to 96.

### 2.2. Phenotypic Characteristics of the Dubska and Hercegovačka Pramenka Sheep

**Figure 1:**
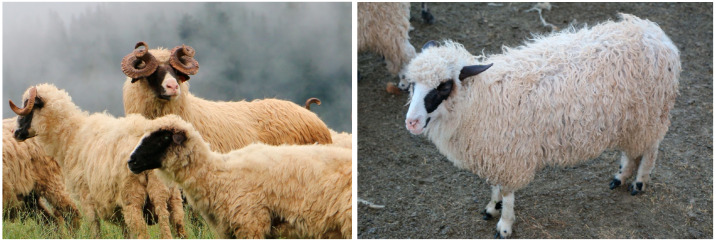
Dubska (**left**) and Hercegovačka (**right**) Pramenka sheep.

Pramenka sheep thrive in diverse ecological zones of Bosnia and Herzegovina, efficiently utilizing available pasture resources with minimal feeding and management requirements. Characterized by predominantly white wool, Pramenka sheep typically exhibit black markings on the head and legs, though these features vary among different strains. Ewes of the Dubska Pramenka strain typically weigh around 50 kg, while rams range from 60 to 65 kg; withers height averages 65–67 cm in females and 72–74 cm in males. Regarding the Hercegovačka strain, ewes weigh around 35 kg, while rams average 45 kg; withers height is approximately 50 cm in females and 55 cm in males. The two investigated strains are shown in Figure 1.

### 2.3. Environmental Data and Temperature–Humidity Index

As previously described in our earlier publication [27], both geographical and meteorological parameters were systematically recorded throughout the duration of the study. Geospatial information related to the investigated areas was gathered using ESRI ArcMap version 10.6.1. This software was also employed for morphological assessments, particularly through the use of a digital terrain model (DTM), which facilitates the reconstruction of landscape features after eliminating surface structures [28]. Meteorological data, including air temperature and relative humidity, were sourced from the Federal Hydrometeorological Institute of Bosnia and Herzegovina (covering Kupres, Vlašić, and Podveležje) and the Republic Hydrometeorological Institute of Banja Luka (for Nevesinje). These climatic variables were utilized to compute the temperature–humidity index (THI). Measurements were collected across the full span of the summer and winter periods as well as on the day of blood sampling and during the five preceding days. Given the comparable environmental conditions with those described in previous studies, the THI calculation was based on the formula proposed by Finocchiaro et al. [29]:

THI = T − [0.55 × (1 − RH)/100] × (T − 14.4),

where T is air temperature (°C) and RH is relative air humidity (%).

### 2.4. Blood Sampling, Total RNA Extraction, and RT-PCR Analysis

Blood samples were collected by jugular vein puncture (*v. jugularis*) using 3 mL EDTA tubes (Ayset^®^ Tube, EDTA 3K). The research included a total of eight thermal stress-related genes: HSP90AA1, HSPA8, HSPA1A, IL-6, IL-10, TNF-α, NOS-3, and SOD-2. Total RNA was isolated from all blood samples using the NucleoSpin™ RNA Kit (Macherey-Nagel™, Dueren, Germany), following manufacturer instructions. Total RNA was reverse transcribed into cDNA using the High-Capacity cDNA Reverse Transcription Kit (Applied Biosystems™, Foster City, CA, USA), according to manufacturer instructions. To obtain the optimal annealing temperature for each primer, primer optimization was performed using PCR Mix Plus Green (A&A Biotechnology^®^, Gdańsk, Poland) and PCRmax Alpha Thermal Cycler (Cole-Palmer^®^, St Neots, UK). PCR primers for sheep, except IL-10, were designed using Primer-BLAST [30]. The forward and reverse primer sequences and optimal annealing temperature are shown in Table 1. Real-time PCR (RT-PCR) was performed using a 7300 Real-Time PCR System (Applied Biosystems, USA) and QuantStudio 5 Real-Time PCR System (Applied Biosystems, USA). The 10 μL reactions consisted of 5 μL SYBR Green Master Mix (Applied Biosystems, USA), 0.25 μL of each forward and reverse primers, 3.5 μL dH_2_O (Millipore Sigma, Burlington, USA), and 1 μL cDNA. Thermal cycling was performed under the following conditions: amplification was started at 95 °C for 5 min, 40 cycles of 95 °C for 40 s, primer annealing (Table 1) for 30 s, and 72°C for 30 s. In the melting temperature analysis, the samples were heated to 95 °C for 15 s, cooled to 54 °C for 1 min, and then heated to 95 °C. Glyceraldehyde 3-phosphate dehydrogenase (GAPDH) was used as the reference gene.

### 2.5. Statistical Analysis

Relative gene expression values were normalized to GAPDH and analyzed with the Pfaffl method [31]. The permutation T-test was applied to compare the differences between two groups using PAST software (version 4.11) [32]. To compare gene expression levels, a chi-square (χ^2^) test (using 2 × 2, 2 × 3, 3 × 3, and 3 × 4 contingency tables) was conducted (MedCalc Software Ltd., Oostende, Belgium). The data were categorized into expression level frequencies based on gene expression values. The ΔCt value for each sample was calculated, and the data were then classified into three expression levels: ΔCt < 0.6—upregulated, ΔCt between 0.6 and 1.5—normal (no change in gene expression), and ΔCt > 1.5—downregulated [33]. These classifications were used as qualitative parameters for the analysis. To further validate the results of the expression level frequencies, several statistical tests were employed, including the Monte Carlo permutation test, Fisher’s exact test, Barnard’s test using PAST software [32], and the difference of proportion hypothesis test (MedCalc Software Ltd., Belgium). A statistical significance level of *p* < 0.05 was applied for all the abovementioned tests.

**Table 1:** Data of applied primers.

Gene	Primer Sequence	Amplicon Size	Annealing Temperature	Source
HSP90AA1	F: CCACTTGGCGGTCAAGCATT	79 bp	54 °C	XM_027957416.1
	R: AGGAGCTCGTCTTGGGACAA			
HSPA8	F: TGGGAAGACTGTTACCAACGCT	78 bp	60 °C	XM_012095633.2
	R: GCATCTTTGGTAGCCTGACGC			
HSPA1A	F: AGGACCTTGTCGTCCAGCAC	64 bp	60 °C	NM_001267874.1
	R: AGTCGATGCCCTCGAACAGG			
IL-10	F: GTCGGAAATGATCCATTTTACCT	80 bp	52 °C	[34]
	R: GTCAGGCCCATGGTTCTCA			
IL-6	F: CCAGGAACGAAAGAGAGCTCCA	78 bp	57 °C	NM_001009392.1
	R: GTGGACTGAAGGCGCTTGTG			
NOS-3	F: CAGTCCCAACAGGACGGGC	80 bp	54 °C	NM_001129901.1
	R: GGCCGGGTCTGCAGTTTCC			
SOD-2	F: AGGCGCTGGAGAAGGGTGAT	79 bp	58 °C	NM_001280703.1
	R: TTGATATGGCCCCCACCGTT			
TNF-α	F: GGAGCCACCACGCTCTTCT	67 bp	60 °C	NM_001024860.1
	R: GGGACTGCTCTTCCCTCTGG			
GAPDH	F: ATGGGCGTGAACCACGAGAA	62 bp	54 °C	NM_001190390.1
	R: GTGCAGGAGGCATTGCTGAC			

## 3. Results

### 3.1. Environmental Data

Climatological and geographical characteristics of the investigated sites are presented in Table 2 and were previously reported in Ohran et al. [27]. The THI values were interpreted using the following classification: values below 22.2 indicate no heat stress; values of 22.2–23.3 suggest moderate heat stress; values of 23.3–25.6 indicate severe heat stress; and values exceeding 25.6 are considered indicative of extreme heat stress [35]. While these thresholds are well-established for heat-related stress, there is no universally accepted standard for defining cold stress based on the THI. However, studies on native Spanish sheep breeds have proposed cold stress thresholds at THI values of 9.8 for the Latxa breed and 10.3 for the Manchega breed [36,37]. Seasonal average THI values (THIavg) did not generally suggest heat stress in the studied regions, with the exception of Podveležje, where mild heat stress was observed throughout the summer months. Due to substantial seasonal variability in air temperature and relative humidity across Bosnia and Herzegovina, relying solely on seasonal averages may obscure the true extent of thermal stress. To address this, we calculated additional THI values using average and maximum temperature and humidity data for the day of blood collection (THIavg-BC and THImax-BC) and for the five days preceding sampling (THIavg-5daysBC and THImax-5daysBC), following the methodology of Salces-Ortiz et al. [38]. These more time-sensitive indices revealed pronounced thermal stress in the Podveležje and Nevesinje regions—areas where the Hercegovačka Pramenka strain is raised—indicating episodes of severe to extremely severe heat stress during the summer. Conversely, in the winter months, most THI calculations indicated cold stress across all sites, with the most extreme values recorded in Vlašić and Kupres, regions where Dubska Pramenka is reared. Moreover, minimum temperature values recorded on the day of blood collection (MiT-BC) and five days prior (MiT-5daysBC) during winter were consistently below the species-specific lower critical temperature, confirming the presence of cold stress across all localities.

### 3.2. Quantitative Analysis of Relative Gene Expression Levels

**Figure 2:**
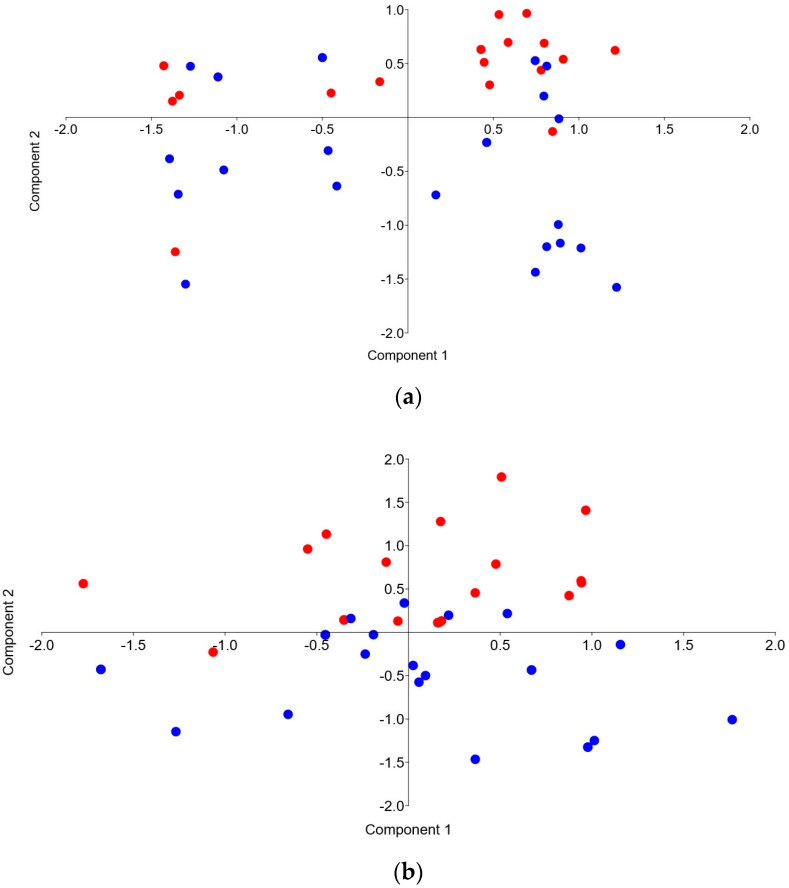
PCA analysis of the expression of all examined genes by strain during the winter (**a**) and summer (**b**) periods of the study. The two sheep strains are labeled with different colors: red—Hercegovačka strain of Pramenka; blue—Dubska strain of Pramenka.

**Figure 3:**
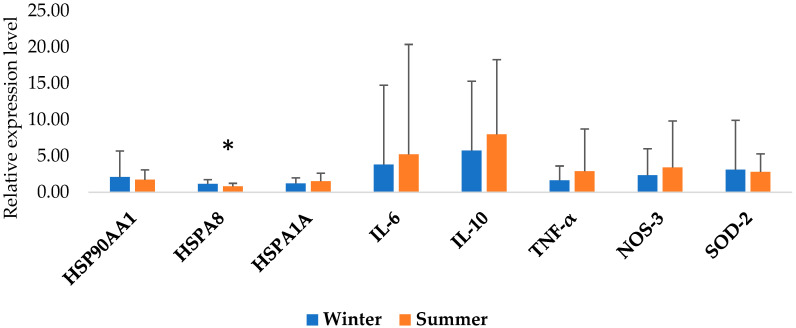
Relative gene expression in total samples between summer and winter periods. All values are presented as $\bar{X}$ ± SD; * *p* < 0.05.

**Figure 4:**
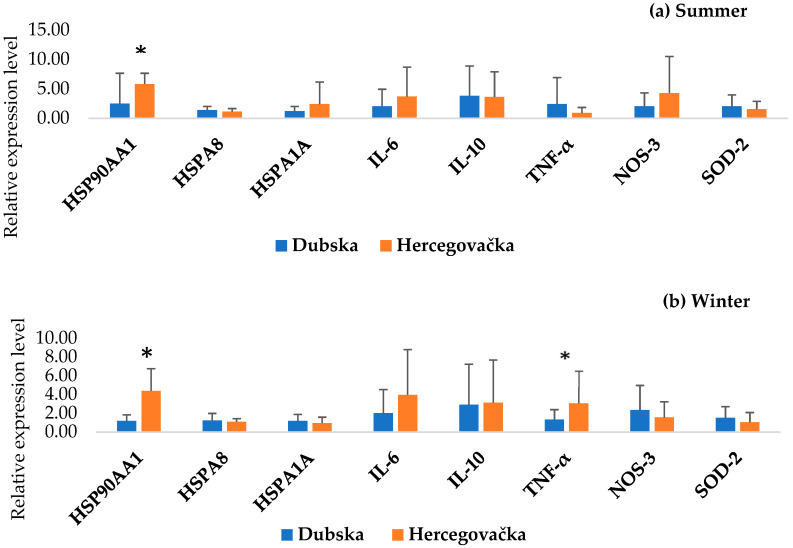
Relative gene expression in summer (**a**) and winter (**b**) periods between Dubska and Hercegovačka strains. All values are presented as $\bar{X}$ ± SD; * *p* < 0.05.

**Figure 5:**
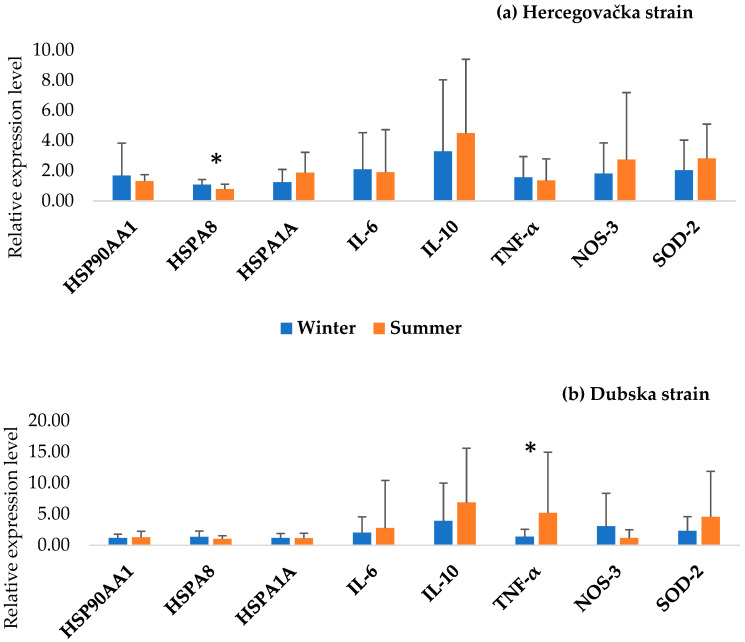
Relative gene expression in Hercegovačka (**a**) and Dubska (**b**) strains in different periods. All values are presented as $\bar{X}$ ± SD; * *p* < 0.05.

**Figure 6:**
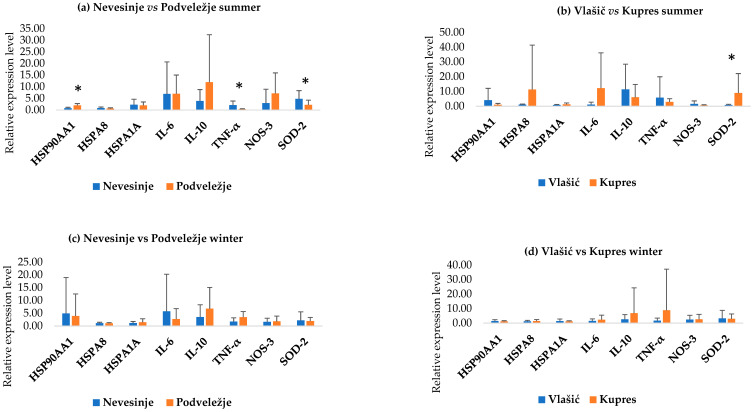
Relative gene expression in summer and winter periods between research areas within examined strains. All values are presented as $\bar{X}$ ± SD; * *p* < 0.05.

**Figure 7:**
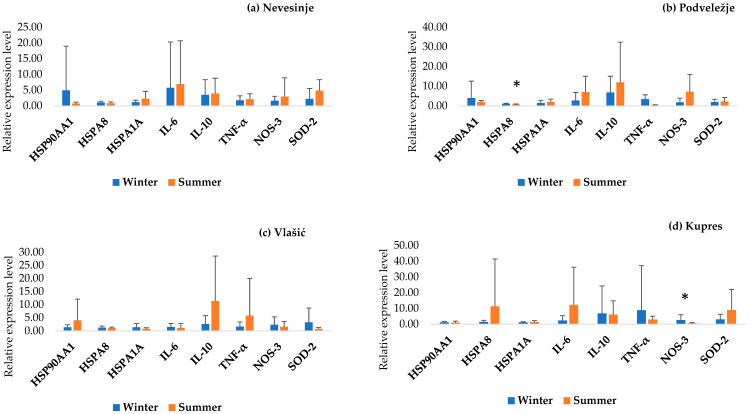
Relative gene expression in summer and winter periods in different research areas. All values are presented as $\bar{X}$ ± SD; * *p* < 0.05.

Figure 2 shows the results of the PCA analysis of all examined genes between the Pramenka strains during both study periods. The relative expression levels of HSP90AA1, HSPA8, HSPA1A, IL-6, IL-10, TNF-α, NOS-3, and SOD-2 are shown in Figure 3, Figure 4, Figure 5, Figure 6 and Figure 7. Statistically significant differences in relative expression levels were predominantly observed for genes of the HSP family and TNF-α. Statistically significant higher expression of HSP90AA1 was observed in the Hercegovačka strain compared to the Dubska strain (Figure 4), both in summer (5.81 vs. 2.47) and in winter (4.38 vs. 1.19), as well as in the Podveležje locality compared to Nevesinje during the summer period (Figure 6a). A lower expression level of the HSPA8 gene was observed in the summer compared to the winter research period in the total sample of both Pramenka strains (0.83 vs. 1.15), in the Hercegovačka strain (0.78 vs. 1.07), as well as in the Podveležje locality (0.61 vs. 1.04) (Figure 3, Figure 5a and Figure 7b). Statistically significantly higher expression of the TNF-α gene was observed in the Hercegovačka strain compared to the Dubska strain during the winter period (3.05 vs. 1.33, Figure 4b). The same gene had higher expression in the summer compared to the winter period in Dubska Pramenka (5.20 vs. 1.38, Figure 5b) and lower expression in the Podveležje locality compared to Nevesinje in the summer period (0.32 vs. 2.07, Figure 6a). The expression level frequencies of the examined genes are shown in Table 3.

## 4. Discussion

The activation of genes indicating thermotolerance following exposure to adverse temperatures is considered to start within just a few hours [39]. Therefore, we based the evaluation of thermal stress on the gene expression on climatological data encompassing the entire summer and winter seasons (THIavg) as well as THI values derived from climatological data on the sampling day and five days before sampling (Table 2). Firstly, the PCA analysis of all examined genes (Figure 2) showed differences in gene expression between the Pramenka strains during both study periods. However, a clearer differentiation is observed when comparing the summer period to the winter period. In the winter period, the first three components explain 64% of the variation, while the PCA conducted for the summer period shows that the first three components account for 62.6% of the variation.

### 4.1. HSP Genes

As widely acknowledged, HSPs are chaperones, mainly acting as cytoprotective proteins. Their main role in thermotolerance is related to the resistance of animals to changing environmental and physiological conditions. Higher expression levels of genes from the HSP family indicate acclimatization and thermal adaptation, as elevated chaperone protein concentrations enhance cellular resilience to heat stress and prevent significant damage, while lower levels increase vulnerability [39,40]. HSP90AA1, the inducible cytoplasmatic isoform of the well-known molecular chaperone HSP90, is activated by unfavorable temperatures [41]. This gene is considered a potential candidate for adaptability and thermotolerance in sheep and could have an important role in sheep adaptation to different climatic and geographic regions [42]. An increased expression level of HSP90AA1 was observed in the Hercegovačka strain compared to the Dubska strain in both examined periods. Moreover, analyzing the expression level frequencies of this gene, it was found that all individuals within the Herzegovinian strain showed increased expression during the summer, while 95.8% of individuals also exhibited elevated expression during the winter. These findings are consistent with global patterns seen in heat-adapted populations. Similar adaptive patterns have been observed in multiple global sheep populations. A study of 24 breeds from Europe, Africa, and Asia found a −660 C/G SNP in the HSP90AA1 promoter region strongly associated with summer expression and climatic variables [42]. In Indian breeds (Marwari, Magra, Chokla, Mandya), HSP90AA1 expression showed the highest levels in arid environments, and overall summer expression was significantly higher than in winter [43]. Furthermore, overexpression resulting from heat has also been noticed in the Spanish Manchega breed [38]. These findings are in accordance with our observations and could be considered a cytoprotective mechanism and an indicator of the adaptive capacity of Hercegovačka Pramenka sheep.

HSP70 is recognized as one of the most consistent biomarkers of cellular stress, and the two major isoforms of this gene are HSPA8 and HSPA1A. HSPA8 supports the essential functions of protein folding, preventing the aggregation of polypeptides, breaking down large protein complexes, and facilitating the translocation of proteins within the cell [44]. Significant differences in the expression of this gene were noted across both strains, with higher expression during winter compared to summer. This trend was also observed within the Hercegovačka strain and the Podveležje site. Such findings could indicate notable thermoresistance, especially against cold stress. This assertion is reinforced by a recent study on thermal adaptability across seventeen sheep breeds [45]. Specifically, the authors identified the heterozygote GA for SNP rs588145625-HSPA8 only in cold-tolerant breeds, including Pramenka. In Munjal sheep, adults and heat-stress susceptible individuals had higher HSP70 expression in both seasons [46]. Similar findings were reported in goats [47], where HSP70 expression during summer was higher in cold-adapted breeds (Gaddi and Chegu) than in heat-adapted ones (Sirohi and Barbari). The authors attributed these variations to breed-specific adaptations to environmental conditions, which may also explain our results, as the Hercegovačka strain is considered to be heat-adapted, while the Dubska strain is associated with cold tolerance. However, Astuti et al. [13] reported heat-stress induced HSP70 expression in the indigenous Hungarian Tsigai. Since the analysis of HSPA8 gene expression frequencies shows predominantly increased expression across most comparison levels in both strains, such results could indicate a conserved cellular response to unfavorable conditions in both strains, especially to cold stress.

### 4.2. Immune-Related Genes

Extreme temperatures outside the animals’ thermoneutral zone directly influence the activation of genes associated with the immune response. Several genes are related to thermal stress and the immune response, including TNF-α, IL-6, and IL-10. These essential and well-known cytokines play multiple roles in the immune response and the regulation pathways of immune-related genes. We observed a statistically significant increase in TNF-α expression levels in the Hercegovačka strain compared to the Dubska strain during the winter season. Furthermore, the Dubska strain showed a significantly higher expression level during the summer compared to the winter period as well as a higher expression during the summer season in the Nevesinje locality compared to Podveležje. Other authors reported similar findings. Shi et al. [48] found that heat-stressed lambs show increased TNF-α gene expression and systemic levels after chronic exposure (e.g., 28 days). Moreover, heat stress induces an increase in circulating leukocytes, TNFα, and IL-6 in feedlot sheep [49], while Merino × Poll Dorset crossbred ewes experienced elevated levels of HSP70, NFκB, and TNFα genes after 7-day heat stress conditions. No statistically significant differences were found in the relative expression levels of IL-6 and IL-10 genes across different seasons, strains, and locations. Rawash et al. [50] found that IL-6 was significantly elevated in Barki sheep during summer in high-temperature zones in Egypt. An in vitro experiment using Comisana dairy ewes PBMCs exposed to hyperthermic conditions revealed elevated IL-6 and suppressed IL-10 under cortisol exposure, showing combined effects of heat and stress hormones on cytokine production [51]. The analysis of gene expression frequencies in both Pramenka strains revealed that nearly all individuals exhibited a statistically significant decrease in TNF-α expression during both research periods (97.2% in the summer and 91.7% in the winter). Moreover, the overall expression patterns indicated reduced IL-6 expression and elevated IL-10 expression throughout. These findings suggest that, under thermal stress conditions, both strains consistently exhibit increased expression of the anti-inflammatory gene IL-10, along with reduced expression of the pro-inflammatory genes IL-6 and TNF-α. Heat stress has been shown to compromise the integrity of the intestinal barrier in several species, facilitating the entry of LPS into the circulatory system and triggering the production of pro-inflammatory cytokines [52,53,54]. Moreover, Chen et al. [55] observed an increase in the pro-inflammatory cytokines IL-6 and TNF-α under heat stress conditions. Such results could indicate that, in both strains, neither heat nor cold stress exerts a pronounced immunosuppressive effect, making them less susceptible to various diseases under thermal stress conditions. Although the association between HSPs and immune responses is well documented, the findings of Siddiqui et al. [56] provide further support for this link. They reported a pattern of increased HSP70 expression accompanied by decreased expression of the pro-inflammatory cytokines IL-6 and TNF-α, suggesting that heat stress may suppress inflammatory responses while enhancing immune regulation. These observations are consistent with our results, which also demonstrated elevated HSPs and IL-10 expression alongside reduced expression of IL-6 and TNF-α under thermal stress conditions in Pramenka sheep. The importance of the immune response during thermal stress was highlighted in the study by Wanjala et al. [10]. Gene ontology enrichment analysis revealed an over-representation of the “Cytokine signaling in the immune system” pathway, underscoring the critical role of the immune response in environmental adaptation.

### 4.3. Oxidative Stress-Related Genes

Oxidative stress causes damage to cellular structures, which can result in a range of disorders. Exposure to environmental stress can elevate ROS production, disrupting the balance between oxidative processes and the antioxidant defense system [57]. Our research focused on two genes associated with oxidative stress: SOD-2 and NOS-3. The antioxidant gene SOD-2 plays a crucial role in mitigating the harmful effects of free radicals and has significant functions in thermotolerance. NO is a signaling molecule with numerous physiological roles, but it is also one of the most potent free radicals with cytotoxic properties. NOS genes can serve as biomarkers for assessing the resistance of sheep and goats to thermal stress [19]. SOD-2 expression was significantly higher at Nevesinje compared to Podveležje during summer, suggesting localized oxidative stress resistance, while NOS-3 was significantly downregulated at Kupres in summer versus winter, and most animals showed reduced NOS-3 overall—potentially indicating adaptive suppression of excess nitric oxide under stress conditions. The results from thermal stress studies in heat-stressed lambs showed decreased SOD-2 expression and enzyme activity, increased lipid peroxidation, and upregulated pro-inflammatory cytokines [48]. Similarly, lambs exposed to outdoor cold experienced suppressed SOD-2/SOD1 gene expression and elevated TNF-α, reflecting weakened antioxidant defenses and compromised immunity [58]. Yadav et al. [21] reported that the expression of genes from the NOS and HSP families follows a similar pattern in goats, similar to our results. HSPA8 plays a key protective role against the production of reactive oxygen species in mitochondria [59], suggesting that the overexpression of HSPA8 may have inhibited the expression of NOS-3 and highlighting the interplay between those genes in our study. The majority of animals had downregulated frequency levels of NOS-3, indicating effective management of oxidative stress in both Pramenka strains. However, further studies on oxidative stress are needed, including a broader range of related genes to better understand the underlying mechanisms.

## 5. Conclusions

The results of this study serve as a strong first indicator of thermal tolerance and considerable genetic adaptability in both investigated strains of Pramenka sheep. Analyzing the expression of all examined HSP genes, the Hercegovačka strain exhibits a more consistent pattern than the Dubska strain, highlighting its greater adaptability to challenging environments. Under thermal stress, both strains exhibited increased expression of the anti-inflammatory IL-10 gene alongside decreased expression of the pro-inflammatory IL-6 and TNF-α genes. These patterns indicate that neither heat nor cold stress imposes substantial immunosuppressive effects, which may contribute to reduced disease susceptibility under extreme temperatures. Additionally, the downregulation of the NOS-3 gene could reflect an enhanced capacity to mitigate oxidative stress caused by adverse thermal conditions. Overall, the findings of this study provide compelling evidence that the examined indigenous Pramenka sheep strains are well-adapted to the harsh environments they traditionally inhabit. These results offer valuable perspectives for developing climate-resilient breeding strategies and for preserving genetic resources that support environmental adaptability of an indigenous breed with limited availability of genetically pure individuals. Although Pramenka sheep are not classified among high-yield breeds intended for intensive production, their greatest potential may lie in their use for genetic improvement of more productive sheep breeds to enhance resilience to climatic stress. Future studies employing RNA sequencing or genome-wide association analyses (GWAS) are warranted to provide deeper insights into the genetic mechanisms of adaptation. Additionally, integrating relevant phenotypic traits will be essential for establishing stronger genotype–phenotype associations. Together, these approaches represent a logical and valuable continuation of the present research.

## Figures and Tables

**Table 2:** Environmental data from the investigated areas [27].

	HERCEGOVAČKA PRAMENKA				DUBSKA PRAMENKA			
	Podveležje		Nevesinje		Vlašić		Kupres	
Geographical data								
Altitude (m)	864		877		1159		1131	
GPS coordinates	43.281656, 17.950202		43.256564, 18.124937		44.311932, 17.538586		43.824834, 17.365428	
Climatological data								
	Summer	Winter	Summer	Winter	Summer	Winter	Summer	Winter
AvT (°C)	20.59	6.76	20.10	5.34	15.08	1.00	15.77	2.11
AvRH (%)	48.73	44.20	49.47	51.72	81.60	61.60	81.53	86.90
THIavg	22.22	4.95	21.62	2.82	15.38	−3.46	16.38	−3.70
AvT-BC	21.06	5.74	19.79	2.00	13.50	5.50	12.77	2.85
MaT-BC	25.66	10.31	25.15	9.75	17.90	8.05	17.79	6.24
MiT-BC	16.91	0.86	14.55	−5.60	10.20	0.80	11.39	−0.06
AvRH-BC	23.17	65.73	58.71	85.17	84.22	97.91	84.06	96.42
RHmax-BC	100.00	62.31	91.45	100.00	100.00	99.60	100.00	98.81
THIavg-BC	21.87	2.66	21.50	−3.74	13.09	0.76	12.03	−3.21
THImax-BC	31.79	8.93	30.50	7.22	19.82	4.61	19.64	1.85
AvT-5daysBC	22.11	3.85	21.69	2.66	15.98	−2.41	16.28	−3.13
MaT-5daysBC	29.76	10.61	31.55	10.55	30.45	7.70	29.39	5.99
MiT-5daysBC	15.56	−0.49	12.00	−3.80	2.85	−10.10	5.11	−8.89
AvRH-5daysBC	54.84	45.23	58.94	65.57	70.43	86.41	70.03	85.85
RHmax-5daysBC	100.00	79.03	94.50	77.57	99.02	99.82	98.93	99.45
THIavg-5daysBC	24.39	1.25	24.01	−1.51	16.58	−10.31	16.99	−11.31
THImax-5daysBC	38.12	8.98	40.37	8.93	39.10	4.06	37.46	1.44

Summer: June–September; Winter: November–February; AvT—average temperature during the entire summer and winter season; AvRH—average relative humidity during the entire summer and winter season; THIavg—THI calculated with average temperature and relative humidity during the entire summer and winter season; AvT-BC—average temperature on the blood collection day; MaT-BC—maximum temperature on the blood collection day; MiT-BC—minimum temperature on the blood collection day; AvRH-BC—average relative humidity on the blood collection day; RHmax-BC—maximum relative humidity on the blood collection day; THIavg-BC—THI calculated with average daily temperature and relative humidity on the blood collection day; THImax-BC—THI calculated with maximum daily temperature and relative humidity on the blood collection day; AvT-5daysBC—average temperature for 5 days before blood collection; MaT-5daysBC—maximum temperature for 5 days before blood collection; MiT-5daysBC—minimum temperature for 5 days before blood collection; AvRH-5daysBC—average relative humidity for 5 days before blood collection; RHmax-5daysBC—maximum relative humidity for 5 days before blood collection; THIavg-5daysBC—THI calculated with average temperature and relative humidity for 5 days before blood collection; THImax-5daysBC—THI calculated with maximum temperature and relative humidity for 5 days before blood collection.

**Table 3:** Expression level frequencies of the examined thermal stress-related genes.

				HERCEGOVAČKA PRAMENKA						DUBSKA PRAMENKA					
		Total of Both Strains		Total within Strain		Nevesinje		Podveležje		Total within Strain		Vlašić		Kupres	
		Summer	Winter	Summer	Winter	Summer	Winter	Summer	Winter	Summer	Winter	Summer	Winter	Summer	Winter
**HSP90AA1**	U	32 (72.7%)	28 (63.6%)	24 (100%)	23 (95.8%)	12 (100%)	12 (100%)	12 (100%)	12 (100%)	8 (38.1%)	6 (26.1%)	5 (41.7%)	2 (18.2%)	3 (33.3%)	4 (33.3%)
	N	6 (13.6%)	12 (27.3%)	0	0	0	0	0	0	7 (33.3%)	13 (56.5%)	4 (33.3%)	7 (63.6%)	3 (33.3%)	6 (50%)
	D	6 (13.6%)	4 (9.1%)	0	1 (4.2%)	0	0	0	0	6 (28.6%)	4 (17.4%)	3 (25%)	2 (18.2%)	3 (33.3%)	2 (16.7%)
**HSPA8**	U	35 (81.4%)	40 (93%)	17 (77.3%)	21 (95.5%)	9 (81.8%)	10 (90.9%)	8 (72.7%)	11 (100%)	18 (87.5%)	19 (90.5%)	12 (100%)	10 (83.4%)	6 (66.7%)	9 (100%)
	N	3 (7%)	2 (4.7%)	2 (9.1%)	1 (4.5%)	0	1 (9.1%)	2 (18.2%)	0	1 (4.8%)	1 (4.8%)	0	1 (8.3%)	1 (11.1%)	0
	D	5 (11.6%)	1 (2.3%)	3 (13.6%)	0	2 (18.2%)	0	1 (9.1%)	0	2 (9.5%)	1 (4.8%)	0	1 (8.3%)	2 (22.2%)	0
**HSPA1A**	U	4 (8.7%)	3 (6.5%)	3 (13%)	1 (4.3%)	2 (16.7%)	0	1 (9.1%)	1 (9.1%)	1 (4.3%)	2 (8.7%)	0	2 (16.7%)	1 (9.1%)	0
	N	19 (41.3%)	17 (37%)	10 (43.5%)	8 (34.8%)	5 (41.7%)	4 (33.3%)	5 (45.5%)	4 (36.4%)	9 (39.1%)	9 (39.1%)	4 (33.3%)	3 (25%)	5 (45.5%)	6 (54.5%)
	D	23 (50%)	26 (56.5%)	10 (43.5%)	14 (60.9%)	5 (41.7%)	8 (66.7%)	5 (45.5%)	6 (54.5%)	13 (56.5%)	12 (52.2%)	8 (66.7%)	7 (58.3%)	5 (45.5%)	5 (45.5%)
**IL-6**	U	6 (14.6%)	3 (7.3%)	5 (25%)	1 (5%)	0	1 (11.1%)	5 (41.7%)	0	1 (4.8%)	2 (9.5%)	0	1 (9.1%)	1 (10%)	1 (8.3%)
	N	0	2 (4.9%)	0	1 (5%)	0	0	0	1 (9.1%)	0	1 (4.8%)	0	1 (9.1%)	0	0
	D	35 (85.4%)	36 (87.8%)	15 (75%)	18 (90%)	9 (100%)	8 (88.9%)	7 (58.3%)	10 (90.9%)	20 (95.2%)	18 (85.7%)	11 (100%)	9 (81.8%)	9 (90%)	11 (91.7%)
**IL-10**	U	38 (84.4%)	25 (55.6%)	21 (87.5%)	14 (58.3%)	11 (91.7%)	9 (75%)	10 (83.3%)	5 (41.7%)	17 (81%)	11 (52.4%)	7 (70%)	4 (40%)	10 (90.9%)	7 (63.6%)
	N	2 (4.4%)	7 (15.6%)	1 (4.2%)	3 (12.5%)	1 (8.3%)	0	0	3 (25%)	1 (4.8%)	4 (19%)	0	2 (20%)	1 (9.1%)	2 (18.2%)
	D	5 (11.1%)	13 (28.9%)	2 (8.3%)	7 (29.2%)	0	3 (25%)	2 (16.7%)	4 (33.3%)	3 (14.3%)	6 (28.6%)	3 (30%)	4 (40%)	0	2 (18.2%)
**TNF-α**	U	1 (2.8%)	1 (2.8%)	0	1 (6.2%)	0	1 (12.5%)	0	0	1 (5%)	0	0	0	1 (12.5%)	0
	N	0	2 (5.6%)	0	0	0	0	0	0	0	2 (10%)	0	1 (8.3%)	0	1 (12.5%)
	D	35 (97.2%)	33 (91.7%)	16 (100%)	15 (93.7%)	8 (100%)	7 (87.5%)	8 (100%)	8 (100%)	19 (95%)	18 (90%)	12 (100%)	11 (91.7%)	7 (87.5%)	7 (87.5%)
**NOS-3**	U	1 (2.3%)	2 (4.5%)	1 (4.5%)	1 (4.5%)	1 (10%)	0	0	1 (8.3%)	0	1 (4.5%)	0	1 (9.1%)	0	0
	N	3 (6.8%)	2 (4.5%)	2 (9.1%)	2 (9.1%)	1 (10%)	1 (10%)	1 (8.3%)	1 (8.3%)	1 (4.5%)	0	0	0	1 (9.1%)	0
	D	40 (90.9%)	40 (90.9%)	19 (86.4%)	19 (86.4%)	8 (80%)	9 (90%)	11 (91.7%)	10 (83.3%)	21 (95.5%)	21 (95.5%)	11 (100%)	10 (90.9%)	10 (90.9%)	11 (100%)
**SOD-2**	U	2 (5.1%)	3 (7.7%)	1 (5%)	1 (5%)	1 (11.1%)	0	0	1 (9.1%)	1 (5.3%)	2 (10.5%)	0	2 (18.2%)	1 (12.5%)	0
	N	10 (25.6%)	9 (23.1%)	5 (25%)	3 (15%)	2 (22.2%)	1 (11.1%)	3 (27.3%)	2 (18.2%)	5 (26.3%)	6 (31.6%)	2 (18.2%)	3 (27.3%)	3 (37.5%)	3 (37.5%)
	D	27 (69.2%)	27 (69.2%)	14 (70%)	16 (80%)	6 (66.7%)	8 (88.9%)	8 (72.7%)	8 (72.7%)	13 (68.4%)	11 (57.9%)	9 (81.8%)	6 (54.5%)	4 (50%)	5 (62.5%)

U—Upregulated, N—Normal, D—Downregulated; Highlighted—statistically significant differences determined by χ^2^ test, Monte Carlo permutation test and Fisher’s exact test (*p* < 0.05).

## Data Availability

None of the data were deposited into an official repository. The data can be provided by the corresponding author upon reasonable request.

## Abbreviations

The following abbreviations are used in this manuscript:

HSP	Heat shock protein
IL	Interleukin
TNF	Tumor necrosis factor
NOS	Nitric oxide synthases
SOD	Superoxide dismutase
RT-PCR	Real-time PCR
THI	Temperature–humidity index
ROS	Reactive oxygen species
SNP	Single-nucleotide polymorphism
DTM	Digital terrain model
EDTA	Ethylenediaminetetraacetic acid
GAPDH	Glyceraldehyde 3-phosphate dehydrogenase
Ct	Threshold cycle
PCA	Principal component analysis
